# GnIH Control of Feeding and Reproductive Behaviors

**DOI:** 10.3389/fendo.2016.00170

**Published:** 2016-12-27

**Authors:** Kazuyoshi Tsutsui, Takayoshi Ubuka

**Affiliations:** ^1^Laboratory of Integrative Brain Sciences, Department of Biology and Center for Medical Life Science, Waseda University, Tokyo, Japan; ^2^Jeffrey Cheah School of Medicine and Health Sciences, Brain Research Institute Monash Sunway, Monash University Malaysia, Bandar Sunway, Malaysia

**Keywords:** gonadotropin-inhibitory hormone, gonadotropin-releasing hormone, gonadotropins, reproduction, reproductive behavior, feeding behavior

## Abstract

In 2000, Tsutsui and colleagues discovered a neuropeptide gonadotropin-inhibitory hormone (GnIH) that inhibits gonadotropin release in birds. Subsequently, extensive studies during the last 15 years have demonstrated that GnIH is a key neurohormone that regulates reproduction in vertebrates, acting in the brain and on the pituitary to modulate reproduction and reproductive behavior. On the other hand, deprivation of food and other metabolic challenges inhibit the reproductive axis as well as sexual motivation. Interestingly, recent studies have further indicated that GnIH controls feeding behavior in vertebrates, such as in birds and mammals. This review summarizes the discovery of GnIH and its conservation in vertebrates and the neuroendocrine control of feeding behavior and reproductive behavior by GnIH.

## Introduction

The discovery of “neurosecretion” in the first half of the last century created neuroendocrinology as a new research field in endocrinology. Scharrer proposed “neurosecretion” as a new concept and suggested that hypothalamic neurons that terminate in the neurohypophysis produce and release neurohormones in the 1920s. This new idea was not accepted by the scientific community easily and criticized strongly. In 1949, however, the concept of “neurosecretion” was established by Bargmann. Subsequently, hypothalamic neuropeptides, such as oxytocin (1) and vasopressin (2), which are secreted from the neurohypophysis, were identified. Harris (3) hypothesized from the histology of hypothalamic neurons that hypothalamic neurons terminating at the median eminence (ME) may produce and release neurohormones into the hypophysial portal system from the ME, and they may regulate anterior pituitary hormones secretion. Subsequently, this hypothesis was demonstrated by the discovery of several important neurohormones from the brain of mammals. Thyrotropin-releasing hormone was discovered by Burgus et al. (4) and Boler et al. (5), whereas gonadotropin-releasing hormone (GnRH) was discovered by Matsuo et al. (6) and Burgus et al. (7). Growth hormone-inhibiting hormone (somatostatin) was discovered by Brazeau et al. (8).

In early 1970s, the groups of Schally (6) and Guillemin (7) discovered a hypothalamic neuropeptide that was later named GnRH, which stimulated the release of luteinizing hormone (LH) as well as follicle-stimulating hormone (FSH) from the anterior pituitary. Thereafter, GnRHs have been identified in other vertebrates (9–12). It was generally accepted that GnRH is the sole hypothalamic neuropeptide that regulates gonadotropin release in vertebrates. However, in 2000, Tsutsui and colleagues discovered gonadotropin-inhibitory hormone (GnIH), a hypothalamic neuropeptide that actively inhibits LH and FSH release in quail, which provides the demonstration of a hypothalamic neuropeptide inhibiting gonadotropin release for the first time in any vertebrate (13).

Studies conducted by Tsutsui and colleagues over 15 years showed that GnIH is conserved in vertebrates, from lampreys to humans and acts as a key neurohormone that regulates reproduction [see Ref. (14–26) for reviews]. In addition, recent studies have shown that GnIH has multiple functions other than the control of reproduction (27, 28). Besides regulating gonadotropin secretion, GnIH further regulates reproductive behavior by changing neurosteroid biosynthesis in the brain (28).

On the other hand, food deprivation inhibits the reproductive axis and sexual motivation. Interestingly, recent studies have further indicated that GnIH controls feeding behavior in vertebrates, such as in mammals and birds [for reviews, see Ref. (25, 26)]. Thus, the last 15 years of GnIH research has contributed to a better understanding of the mechanism of neuroendocrine regulation of feeding and reproductive behaviors as well as reproduction [for reviews, see Ref. (16–26, 29)].

Herein, this review summarizes the discovery of GnIH and its conservation in vertebrates and highlights our current understanding of the neuroendocrine control of feeding and reproductive behaviors by GnIH.

## Discovery of GnIH and Its Conservation in Vertebrates

### Discovery of GnIH

The discovery of “neurosecretion” led to create neuroendocrinology. In addition, recent discoveries of novel neuropeptides regulating reproductive physiology have expanded the horizons of this new research field in endocrinology. One of such discoveries was that of GnIH from a search for a novel neuropeptide that regulates pituitary hormones release in the avian brain (13).

**Figure 1:**
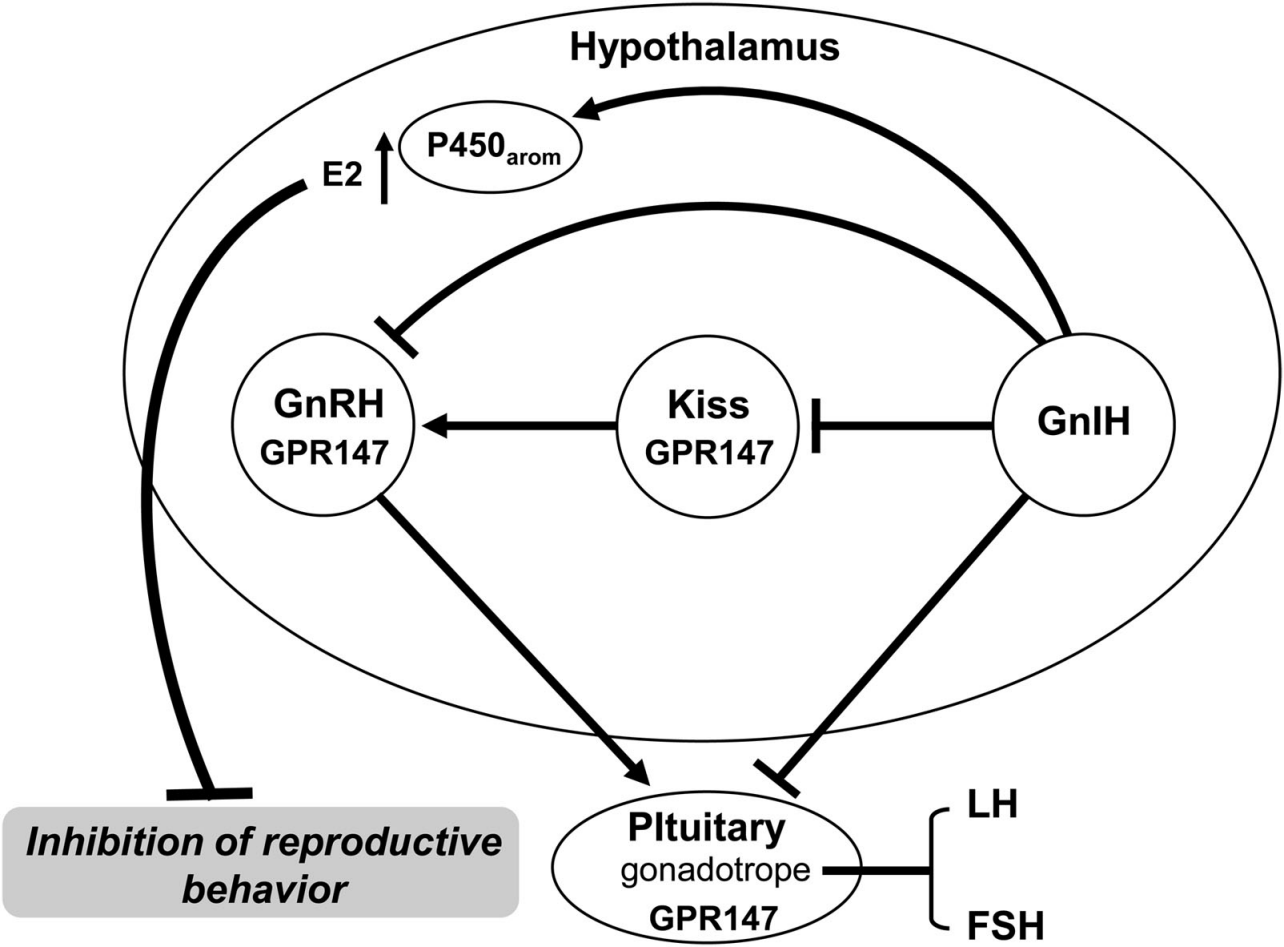
**Gonadotropin-inhibitory hormone (GnIH) control of reproduction and reproductive behavior**. GnIH participates in the control of reproduction and reproductive behavior in birds and mammals. GnIH neurons project to the median eminence to inhibit gonadotropin synthesis and release *via* GnIH receptor (GPR147) expressed in gonadotropes. GnIH neurons also project to gonadotropin-releasing hormone (GnRH) neurons that express GPR147 and inhibit GnRH neuronal activity. Thus, GnIH inhibits gonadotropin synthesis and release by decreasing the activity of GnRH neurons as well as directly inhibiting pituitary gonadotrope function. GnIH neurons also project to kisspeptin (Kiss) neurons that express GPR147 in mammals. GnIH neurons further project to P450 aromatase neurons and stimulate aromatase activity to produce neuroestrogen (E2) that inhibits reproductive behavior. See the text for details.

Gonadotropin-inhibitory hormone is a new hypothalamic neuropeptide that possesses a C-terminal sequence Arg-Phe-NH_2_ (RFamide peptide), which was isolated by high-performance liquid chromatography as well as competitive enzyme-linked immunosorbent assay in the Japanese quail brain (13). RFamide peptide was first identified in the late 1970s by Price and Greenberg who identified a peptide that has a sequence of Phe-Met-Arg-Phe-NH_2_ from the ganglia of the venus clam and named FMRFamide (30). Subsequently, numerous RFamide peptides that act as neuromodulators, neurotransmitters, and peripheral hormones had been identified in invertebrates species. Importantly, immunohistochemical studies suggested that vertebrates also possess hypothalamic RFamide peptide(s) that may act on the anterior pituitary and regulate pituitary hormones secretion (31, 32). Tsutsui and colleagues made a breakthrough by discovering a novel RFamide peptide in 2000. The peptide had a sequence of Ser-Ile-Lys-Pro-Ser-Ala-Tyr-Leu-Pro-Leu-Arg-Phe-NH_2_ (SIKPSAYLPLRFamide) and actively inhibited gonadotropin release from cultured quail anterior pituitary (Tables 1 and 2). This discovery provided the first demonstration of an inhibitory hypothalamic neuropeptide on gonadotropin release, which was not shown in any vertebrate (13). Given its biological action, this peptide was named GnIH (13) (Figure 1). In birds, GnIH neuronal cell bodies are located in the paraventricular nucleus (PVN) and terminals are found in the ME (13). The C-terminal of GnIH peptide is identical to LPLRFamide peptide of chicken (33), which may be a degraded C-terminal fragment of GnIH [for reviews, see Ref. (16, 21, 22)]. The GnIH precursor protein cDNA was cloned in quail (34) as well as other avian species [for reviews, see Ref. (16, 21, 22)]. The GnIH precursor protein encodes one GnIH and two GnIH-related peptides (GnIH-RP-1 and GnIH-RP-2) that possess an LPXRFamide (X = L or Q) motif at their C-terminus in all avian species investigated. Mature form of GnIH was also identified in starlings (35), zebra finches (36), as well as chicken (37) in birds. Quail GnIH-RP-2 was also identified (34) (Tables 1 and 2).

**Table 1:** **Molecular structure and behavioral actions of mature gonadotropin-inhibitory hormone (GnIH) peptides identified in birds and mammals**.

Vertebrates	Molecular structure of mature GnIH peptides	Receptor and cell signaling of mature GnIH peptides	Behavioral actions of mature GnIH peptides
Mammals	MPHSFANLPLRFa (*human GnIH*; *RFRP-1*) VPNLPQRFa (*human GnIH*; *RFRP-3*) SGRNMEVSLVRQVLNLPQRFa (*macaque GnIH*; *RFRP-3*) SLTFEEVKDWAPKIKMNKPVVNKMPPSAANLPLRFa (*bovine GnIH*; *RFRP-1*) AMAHLPLRLGKNREDSLSRWVPNLPQRFa (*bovine GnIH*; *RFRP-3*) ANMEAGTMSHFPSLPQRFa (*rat GnIH*; *RFRP-3*) SPAPANKVPHSAANLPLRFa (*Siberian hamster GnIH*; *RFRP-1*) TLSRVPSLPQRFa (*Siberian hamster GnIH*; *RFRP-3*)	GPR147 is the primary and GPR74 is the secondary receptor for human GnIH (*human*). Activation of GPR147 suppresses cAMP production (*human*). Mammalian GnIHs (RFRPs) suppress adenylate cyclase /cAMP/ protein kinase A-dependent extracellular signal-regulated kinase phosphorylation (*mouse gonadotrope cell line*). Gonadotropin-releasing hormone (GnRH) neurons express GPR147 (*Siberian hamsters*). GnRH neurons express GPR147 mRNA (*mice*). Kiss1 neurons express GPR147 and GPR74 mRNAs (*mice*). Gonadotropes express GPR147 mRNA (*human*).	Central administration of mammalian GnIH (RFRP-3) decreases male sex behavior (*rats*). Central administration of avian GnIH reduces sexual motivation and vaginal scent marking (*female hamsters*). Central administration of mammalian GnIHs (RFRPs) induces anxiety-related behavior (*rats*). Central administration of mammalian GnIH (RFRP-3) increases food intake (*male rats, sheep*). Bilateral intraamygdalar administration of mammalian GnIH (RFRP-3) decreases food intake (*male rats*). Bilateral intraamygdalar administration of mammalian GnIH (RFRP-1) has positive rewarding–reinforcing properties (*male rats*).

Birds	SIKPSAYLPLRFa (*quail GnIH*) SSIQSLLNLPQRFa (*quail GnIH-RP-2*) SIRPSAYLPLRFa (*chicken GnIH*) SIKPFANLPLRFa (*European starling GnIH*) SIKPFSNLPLRFa (*zebra finch GnIH*)	GPR147 is the primary receptor for avian GnIH (*quail, chicken*). Activation of GPR147 suppresses cAMP production (*chicken*). GnRH1 and GnRH2 neurons express GPR147 mRNA (*starlings*). GPR147 is colocalized with luteinizing hormone β or follicle-stimulating hormone β mRNA in the pituitary (*chicken*).	Central administration of avian GnIH inhibits copulation solicitation (*female white-crowned sparrows*). Avian GnIH RNA interference reduces resting time, spontaneous production of complex vocalizations, and stimulated brief agonistic vocalizations (*white-crowned sparrows*). Central administration of avian GnIH decreases socio-sexual behavior (*male quail*). Central administration of avian GnIH, GnIH-RP-1 and GnIH-RP-2 stimulates food intake (*chicks*). Central administration of avian GnIH stimulates feeding (*adult Pekin drakes*).

*Names of the investigated species or organism are shown in the parenthesis*.

**Table 2:** **Molecular structure of gonadotropin-inhibitory hormone (GnIH) peptides in chordates**.

Chordates	Peptide name	Molecular structure
Mammals	*Human RFRP-1*	MPHSFAN**LPLRFa**
	*Human RFRP-3*	VPN**LPQRFa**
	*Macaque RFRP-3*	SGRNMEVSLVRQVLN**LPQRFa**
	*Bovine RFRP-1*	SLTFEEVKDWAPKIKMNKPVVNKMPPSAAN**LPLRFa**
	*Bovine RFRP-3*	AMAHLPLRLGKNREDSLSRWVPN**LPQRFa**
	*Rat RFRP-3*	ANMEAGTMSHFPS**LPQRFa**
	*Siberian hamster RFRP-1*	SPAPANKVPHSAAN**LPLRFa**
	*Siberian hamster RFRP-3*	TLSRVPS**LPQRFa**

Birds	*Quail GnIH*	SIKPSAY**LPLRFa**
	*Quail GnIH-RP-2*	SSIQSLLN**LPQRFa**
	*Chicken GnIH*	SIRPSAY**LPLRFa**
	*European starling GnIH*	SIKPFAN**LPLRFa**
	*Zebra finch GnIH*	SIKPFSN**LPLRFa**

Reptiles	*Red-eared slider GnIH*	SIKPVAN**LPLRFa**
	*Red-eared slider GnIH-RP-1*	STPTVNKMPNSLAN**LPLRFa**
	*Red-eared slider GnIH-RP-2*	SSIQSLAN**LPQRFa**

Amphibians	*Bullfrog GRP/R-RFa*	SLKPAAN**LPLRFa**
	*Bullfrog GRP-RP-1*	SIPN**LPQRFa**
	*Bullfrog GRP-RP-2*	YLSGKTKVQSMAN**LPQRFa**
	*Bullfrog GRP-RP-3*	AQYTNHFVHSLDT**LPLRFa**
	*Red-bellied newt LPXRFa-1*	SVPN**LPQRFa**
	*Red-bellied newt LPXRFa-2*	MPHASAN**LPLRFa**
	*Red-bellied newt LPXRFa-3*	SIQPLAN**LPQRFa**
	*Red-bellied newt LPXRFa-4*	APSAGQFIQTLAN**LPQRFa**
Teleost fishes	*Goldfish LPXRFa-3*	SGTGLSAT**LPQRFa**

Agnathans	*Sea lamprey LPXRFa-1a*	SGVGQGRSSKTLFQ**PQRFa**
	*Sea lamprey LPXRFa-1b*	AALRSGVGQGRSSKTLFQ**PQRFa**
	*Sea lamprey LPXRFa-2*	SEPFWHRTR**PQRFa**

Protochordates	*Amphioxus PQRFa-1*	WDEAWR**PQRFa**
	*Amphioxus PQRFa-2*	GDHTKDGWR**PQRFa**
	*Amphioxus PQRFa-3*	GRDQGWR**PQRFa**

*Only mature endogenous peptides structurally determined by high-performance liquid chromatography and mass spectrometry are shown. Characteristic C-terminal sequences for GnIH peptides, -LPXRFamide (X = L or Q) sequences, are shown in bold*.

Gonadotropin-inhibitory hormone is considered to be a key neurohormone that inhibits avian reproduction as GnIH was shown to inhibit gonadotropin secretion in most of avian species that was studied [for reviews, see Ref. (16, 21, 22)] (Figure 1). To demonstrate the biological action of GnIH, Ubuka et al. (38) administered GnIH to mature male quail *in vivo* chronically. Chronic GnIH treatment decreases the concentration of plasma LH and testosterone and the expressions of common α, LHβ, and FSHβ subunit mRNAs. Furthermore, GnIH treatment induces apoptosis in testicular cells and decreases the diameter of seminiferous tubules in mature birds (38). Further, GnIH treatment also suppresses normal testicular growth and the increase in testosterone concentration in immature birds (38). Based on extensive studies, it appears that GnIH suppresses the development of the gonad and its maintenance by decreasing synthesis and release of gonadotropin in birds (Figure 1).

### Conservation of GnIH in Vertebrates

To demonstrate GnIH conservation in other vertebrates, GnIHs were further identified in the hypothalamus of mammals and primates (39–43). The identified mammalian GnIH peptides possess C-terminal LPXRFamide (X = L or Q) as a common motif, as in avian GnIH and GnIH-RPs [for reviews, see Ref. (16, 17, 21–25)] (Tables 1 and 2). GnIH peptides were named LPXRFamide peptides based on the structure of their C-terminal. Mammalian GnIHs are also named RFamide-related peptide 1 and 3 (RFRP-1 and -3) (Tables 1 and 2). Administration of avian GnIH to Syrian hamsters centrally or peripherally inhibits LH release (40). It was shown that central administration of Siberian hamster GnIHs (RFRP-1 and -3) to Siberian hamsters also inhibits LH release (43). Centrally administered rat GnIH (RFRP-3) also inhibits LH release in rats (44) as well as GnRH-elicited LH release (45, 46). GnIH (RFRP-3) also inhibits GnRH-elicited gonadotropin synthesis and release and reduces LH pulse amplitude in ovine (47, 48) as well as bovine (49). Since human GnIH (RFRP-3) has the same structure as ovine GnIH (RFRP-3) (42), the biological action of human/ovine GnIH (RFRP-3) was investigated in the ovine pituitary. It was clearly shown that human/ovine GnIH (RFRP-3) inhibits GnRH-elicited secretion of LH and FSH (47), demonstrating that human/ovine GnIH inhibits synthesis and release of gonadotropin as well as GnRH-elicited gonadotropin secretion like avian GnIH [for reviews, see Ref. (16, 17, 21–26)] (Figure 1).

Tsutsui and colleagues further identified GnIH peptides in the brains of reptiles, amphibians, and fish. All of the identified or putative GnIHs also had a characteristic C-terminal LPXRFamide (X = L or Q) motif in these species, which avian and mammalian GnIHs have (50–57) (Table 2). Accordingly, GnIH peptides are produced in the brains of vertebrates across fish to humans [see Ref. (14–25) for reviews]. Three GnIHs, gfLPXRFa-1, -2, and -3 are encoded in goldfish GnIH precursor cDNA (52) (Table 2). It was shown that gfLPXRFa-1, -2, and -3 have inhibitory as well as stimulatory effects on synthesis and release of gonadotropin, which may depend on the reproductive phase (58–61). It was also shown that zfLPXRF-3, zebrafish GnIH, inhibits gonadotropin release (62).

As mentioned above, GnIH peptides were identified in gnathostomes from humans to fish. However, in the most ancient lineage of vertebrates, agnathans, the presence of GnIH peptide was not known (63). Accordingly, Tsutsui and colleagues searched for agnathan GnIH. Osugi et al. (64) cloned sea lamprey GnIH precursor cDNA. Three mature GnIH peptides were identified from the sea lamprey brain using immunoaffinity purification as well as mass spectrometry (64) (Table 2). The identified lamprey GnIHs have a C-terminal PQRFamide motif in common (64). Lamprey GnIH neuronal cell bodies exist in the hypothalamus and their immunoreactive fibers project to GnRH3 neurons (64). A lamprey GnIH peptide increases the expressions of lamprey GnRH3 and gonadotropin β mRNA. Lamprey GnIH may also act on GnRH3 neurons and stimulate gonadotropin β expression in the pituitary (64). Accordingly, GnIH may be a stimulatory neuropeptide in agnathans and changed its function to be an inhibitory neuropeptide during the evolution of vertebrates.

### Evolutionary Origin of GnIH

Most GnIH peptides have C-terminal structure of LPXRFamide (X = L or Q), within a member of the RFamide peptide family [see Ref. (14–25) for reviews]. The neuropeptide FF (NPFF; PQRFamide peptide) group is also a member of the RFamide peptide family as NPFF peptides have a C-terminal PQRP motif [for reviews, see Ref. (14–17)]. Since the C-terminal structure of GnIH peptides is similar to that of NPFF peptides, further clarification of the NPFF peptide gene was warranted in agnathans. Tsutsui and colleagues accordingly identified the precursor cDNAs of NPFF peptides from the brains of lamprey and hagfish (65, 66). The phylogenetic analysis showed that agnathans genes encode GnIH and NPFF (65, 66). The identified agnathan NPFF peptides had the same C-terminal PQRFamide motif that also exists in agnathan GnIH peptides (65, 66). Based on these findings, Tsutsui and colleagues hypothesized that GnIH and NPFF genes derive from their ancestral gene of protochordates.

To demonstrate this hypothesis, Tsutsui and colleagues further cloned a precursor cDNA of PQRFamide peptide, which encodes three putative PQRFamide peptides in amphioxus (67). Mature forms of these three endogenous amphioxus PQRFamide peptides were identified by immunoaffinity purification as well as mass spectrometry (67) (Table 2). Phylogenetic analysis suggested that the amphioxus PQRFamide peptide precursor occurred before the divergence of GnIH and NPFF groups in vertebrates (67). Importantly, the conserved synteny region exists around the loci of the amphioxus PQRFamide peptide gene, as well as GnIH and NPFF gene in vertebrates (67). Namely, the amphioxus PQRFamide peptide gene is located close to the HOX cluster, whereas the GnIH and NPFF genes in vertebrates are located close to the HOXA and HOXC clusters, respectively. These results suggest that the GnIH and NPFF genes may duplicated by whole-genome duplications (67). The amphioxus PQRFamide peptide gene is therefore considered to be related to the ancestor of the GnIH and NPFF genes (67, 68). Thus, it is possible that the GnIH and NPFF genes diverged from the ancestral gene in protochordate during vertebrate evolution by whole-genome duplication.

## Control of GnIH Expression and Mode of GnIH Action

### Control of GnIH Expression by Environmental and Internal Factors

Studying the mechanisms controlling GnIH expression is important to understand the physiological role of GnIH. Stress inhibits reproduction in vertebrates (69). Calisi et al. (70) examined the effect of capture-handling stress on GnIH expression in house sparrows to investigate if stress changes GnIH expression. The number of GnIH-positive neurons increased in birds sampled in fall compared with birds in spring, and the numbers of GnIH-positive neurons increased in spring birds by capture-handling stress (70). These findings indicate that stress influences GnIH expression during the breeding season (70). Thus, stress may inhibit reproductive function in birds through GnIH neurons. In mammals, it was also found that acute and chronic immobilization stress both upregulate GnIH expression in the dorsomedial hypothalamic area of rats with a decrease in the activity of hypothalamic–pituitary–gonadal axis (HPG axis) (71). This stress-induced increase in GnIH expression is abolished by adrenalectomy (71). Immunohistochemical analysis further revealed that GnIH neurons express glucocorticoid receptor (GR) (71), suggesting that adrenal glucocorticoids directly act on GnIH neurons *via* GR to increase GnIH expression. Thus, it is considered that GnIH serves as an important stress integrator in the suppression of the reproductive axis in vertebrates.

Son et al. (72) found that GnIH neurons in the PVN express GR mRNA in quail, which suggests that glucocorticoids can directly control GnIH transcription in birds. In addition, corticosterone (CORT) treatment increases expression of GnIH precursor mRNA in the quail diencephalon (72). Furthermore, Son et al. (72) examined the transcription mechanism of GnIH gene by CORT using rHypoE-23 cells, a rat hypothalamic GnIH-expressing neuronal cell line. It was shown that rHypoE-23 cells express GR mRNA and CORT increases expression of GnIH mRNA (72). In addition, CORT stimulates the recruitment of GR to the GC response element in the promoter region of rat GnIH, supporting the idea that CORT induces GnIH expression *via* GR in GnIH neurons (72). It thus appears that stress reduces gonadotropin release by increasing GnIH expression in GnIH neurons.

It is thought that photoperiodic mammals utilize the annual changes in the nocturnal melatonin secretion to regulate reproductive activities (73). In photoperiodic birds, melatonin participates in the regulation of seasonal reproductive processes, such as gonadotropin secretion and gonadal activity (74–77), despite the dogma that seasonal changes in melatonin secretion is not used to time reproductive activities in birds (78, 79). Tsutsui and colleagues therefore investigated whether melatonin is involved in the regulation of GnIH expression in quail, a highly photoperiodic avian species (80). Melatonin is mostly produced in the pineal gland and eyes in quail (81). Ubuka et al. (80) found that pinealectomy together with orbital enucleation (Px + Ex) decreases GnIH precursor mRNA expression and GnIH peptide concentration in the quail brain. Melatonin administration increases GnIH precursor mRNA expression and GnIH peptide concentration in the quail brain (80). Importantly, a melatonin receptor subtype Mel_1c_ is expressed in GnIH neurons in the PVN (80). Chowdhury et al. (82) further demonstrated that melatonin increases GnIH release as well as GnIH expression in quail. GnIH release increases under short day (SD), when nocturnal secretion of melatonin is long (82). Importantly, GnIH release is negatively correlated with plasma LH concentration with their diurnal changes in quail (82). Based on these findings, it is considered that melatonin synthesized in the pineal gland and eyes acts directly on GnIH neurons *via* Mel_1c_ to induce GnIH expression and release in birds (24, 80, 82).

In contrast to birds, melatonin reduces GnIH expression in Syrian and Siberian hamsters, photoperiodic mammals (43, 83, 84). GnIH precursor mRNA levels as well as the number of GnIH cell bodies decrease in sexually inactive Syrian and Siberian hamsters kept under SD photoperiods, compared with sexually active animals kept in long day (LD) photoperiods. These photoperiodic changes in GnIH expression disappear in Px hamsters; however, melatonin injections to LD hamsters reduce GnIH expression to SD levels (43, 83). Seasonal GnIH expression patterns were similar in European and Turkish hamsters (85, 86) and the semi-desert rodent, Jerboa (87). Although these results suggest a role for GnIH in seasonal breeding, it is inconsistent with the seasonal reproductive control model. Hamsters may require abundant GnIH to suppress GnRH in LD, whereas high level of GnIH is unnecessary in SD hamsters of regressed reproductive axis. Ubuka et al. (43) clearly showed that GnIH administration suppresses gonadotropin secretion in LD, but stimulates it in SD, suggesting the role of GnIH to fine tune the reproductive axis according to different photoperiods. In sheep (88, 89) and rats (90), there are also reports showing that GnIH expression is controlled by season and melatonin. Accordingly, GnIH expression is modulated by melatonin in mammals, as in birds.

In addition to stress and photoperiod that are important environmental factors, social environment may also influence GnIH expression because reproductive physiology and behavior can vary between individuals even in the same natural environment. To determine this possibility, Calisi et al. (91) investigated the effect of competition for mating on GnIH. The opportunities of nesting for European starlings pairs were restricted and GnIH precursor mRNA and GnIH content in the brain were investigated. Birds that occupied nest boxes had fewer GnIH cells than birds without nest boxes. These results suggest a role of GnIH in the modulation of reproductive function in response to the social environment (91).

There is evidence that female bird presence and copulation decrease plasma T concentrations in male quail rapidly (92, 93). Tsutsui and colleagues therefore examined the mechanism of how social stimuli change reproductive physiology and behavior. Recently, Tobari et al. (27) first found that the release of norepinephrine (NE) increases in the PVN rapidly in male quail when viewing a conspecific female. Likewise, GnIH precursor mRNA increases in the PVN associated with plasma LH decrease in male when viewing a female. Tobari et al. (27) then demonstrated a link between these two changes by showing that NE applied to diencephalic tissue blocks stimulates GnIH release *in vitro*. Tobari et al. (27) further found that GnIH neurons are innervated by noradrenergic fibers and express α2A-adrenergic receptor mRNA in male quail. Accordingly, it is considered that female presence increases NE release in the PVN that stimulates GnIH release, which suppress LH release in male quail (27).

### Mode of GnIH Action on Gonadotropin Secretion

To reveal the mode of GnIH action on the secretion of gonadotropin, Tsutsui and colleagues characterized the receptor for GnIH in quail. The GnIH receptor, GPR147, identified in quail is a member of the G-protein coupled receptor (GPCR) superfamily (94), which is also named neuropeptide FF receptor 1 (NPFF1). The COS-7 cells membrane fraction transfected with GnIH receptor cDNA specifically binds GnIH and GnIH-RPs at high affinities (94). By contrast, non-amidated GnIH cannot bind the GnIH receptor. Accordingly, the C-terminal LPXRFamide (X = L or Q) motif is critical for its binding to GnIH receptor (94). GnIH receptor cDNA was also cloned in chicken (95). The GnIH receptor exists in gonadotropes in the pituitary, and GnIH acts on gonadotropes directly to reduce gonadotropin synthesis and release in birds [for reviews, see Ref. (16, 17, 21–26, 29)] (Figure 1). Ultrastructural studies of GnIH neurons to explore the neurosecretory nature are progressing. GnIH neurons further project to GnRH1 neurons expressing GnIH receptor in birds (35, 96–98) (Figure 1). Accordingly, it appears that in birds GnIH acts on gonadotropes as well as GnRH1 neurons to inhibit gonadotropin synthesis and release [see Ref. (16, 17, 21–26, 29) for reviews] (Figure 1).

In mammals, Hinuma et al. (99) identified a mammalian GnIH (RFRP) specific receptor that is identical to GPR147, and it was named OT7T022. In the same year, Bonini et al. (100) reported two different GPCRs for NPFF and named NPFF1 (same as GPR147) and NPFF2 (same as GPR74). As mentioned previously, NPFF has a C-terminal PQRFamide motif and is involved in pain modulation. GnIH (LPXRFamide peptide) and NPFF (PQRFamide peptide) genes may have evolved from the ancestral gene by gene duplication (64, 67, 68). GPR147 and GPR74 are thought to be paralogous (101). GnIH binds GPR147 at higher affinity, whereas NPFF binds GPR74 at high affinity (100, 102, 103). Thus, GPR147 (NPFF1, OT7T022) is considered to be the primary GnIH receptor.

To demonstrate the mode of GnIH action on the gonadotropes, Tsutsui and colleagues investigated GnIH receptor signaling pathways in LβT2 cells, a mouse gonadotrope cell line, which expresses GnIH receptor mRNA (104). In LβT2 cells, mouse GnIHs effectively reduce cAMP production and phosphorylation of extracellular signal-regulated kinase (ERK) induced by GnRH (104). Furthermore, mouse GnIHs reduce GnRH-induced LHβ expression and LH release (104). Adenylate cyclase (AC) and protein kinase A (PKA) inhibitors suppress the stimulatory effect of GnRH on gonadotropin expression (104). Thus, mouse GnIH reduces GnRH-stimulated gonadotropin secretion by specifically interfering with GnRH actions *via* a AC/cAMP/PKA-dependent ERK pathway (104).

Kisspeptin (Kiss) encoded by the *Kiss-1* gene is also a newly identified neuropeptide in mammals, following the discovery of GnIH. In mammals, Kiss possesses a C-terminal RFamide or RYamide motif. In contrast to GnIH, Kiss stimulates GnRH neurons and upregulates the HPG axis in mammals (105–108). Importantly, GnIH neurons project to GnRH1 neurons and Kiss neurons that express GnIH receptor (16, 17, 21–26, 109) (Figure 1). Therefore, GnIH may act on GnRH1 neurons and Kiss neurons to regulate their activities (16, 17, 21–26, 109) (Figure 1). Furthermore, GnIH neurons project not only to GnRH1 neurons but also to GnRH2 neurons and many other neurons in the brain, suggesting multiple actions of GnIH [for reviews, see Ref. (16, 17, 21–26)].

Because GnIH neurons express ERα and respond to E2 administration with c-Fos expression in rodents, Kriegsfeld et al. (40) suggested that GnIH is involved in estrogen feedback to GnRH neurons. Gibson et al. (110) showed that when endogenous E2 concentration is high at the time of the GnRH/LH surge the activity of GnIH neurons is low, suggesting that high E2 removes its negative feedback effect by decreasing the negative effect of RFRP-3 on GnRH neurons in female hamsters. It was further shown that the suprachiasmatic nucleus (SCN) projects to a large population of GnIH neurons, and SCN, GnRH, and GnIH neuronal activities are coordinated with ovulation (110). Molnár et al. (111) investigated the involvement of GnIH neurons in E2 feedback in mice. GnIH mRNA expression was compared in ovariectomized mice with and without E2 replacement. Subcutaneously, administered E2 for 4 days significantly suppressed GnIH mRNA expression. Salehi et al. (112) measured GnIH gene expression during the estrous cycle in the rat hypothalamus and found that GnIH mRNA expression during proestrus is lower when endogenous E2 concentration is the highest than the diestrus phase (112). Furthermore, Jørgensen et al. (113) showed that c-Fos-positive RFRP-1-ir neurons increase in diestrus when endogenous E2 concentration is lower as compared with proestrus in the female rat brain (113). Accordingly, downregulation of GnIH expression by estrogen may be the mechanism of estrogen positive feedback to GnRH/LH release at least in female rodents [see Ref. (114, 115) for reviews].

### Direct Control of Reproduction by Gonadal GnIH

Accumulated findings indicate that GnIH is a key neuropeptide in the control of reproduction, by decreasing the activity of GnRH1 neurons in the hypothalamus to reduce gonadotropin synthesis and release and directly decreasing the activity of pituitary gonadotropes, resulting in the suppression of spermatogenesis and gonadal steroid secretion. In addition to the central actions of GnIH, direct control of reproduction by gonadal GnIH is becoming clear [for reviews, see Ref. (16, 17, 21–26, 28, 29)]. GnIH and GnIH receptor are expressed in steroidogenic and germ cells in the gonads of birds and mammals, possibly acting in autocrine or paracrine mechanisms to suppress production of gonadal steroid and germ cell differentiation and maturation (116–122). There are also several reports in songbirds, showing that gonadal GnIH is directly regulated by melatonin, metabolic challenge, and stress according to season (123–125).

## GnIH Control of Feeding Behavior

Importantly, several lines of evidence indicate that GnIH not only regulates neuroendocrine functions but also behavior. Animals use photoperiod to time breeding according to maximal food availability anticipated in environments where energy availability changes according to season (73). Reproduction is temporarily inhibited when food become scarce during the breeding season (126). Reproductive function and sexual motivation are inhibited by deprivation of food and other metabolic stress (127–131). Therefore, it is considered that GnIH may control neural feeding circuits by transferring metabolic information to the HPG axis [for reviews, see Ref. (25, 26)].

**Figure 2:**
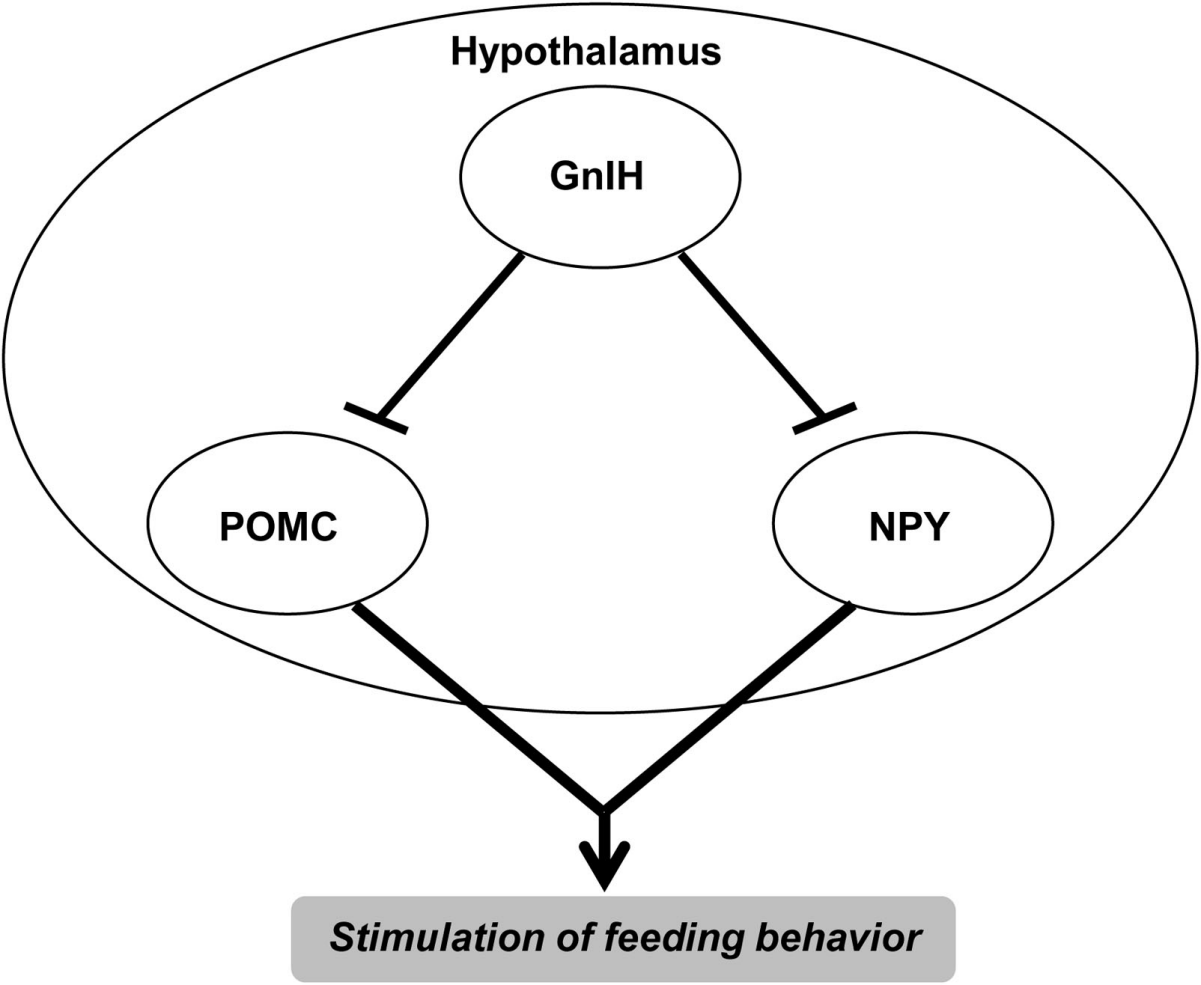
**Gonadotropin-inhibitory hormone (GnIH) control of feeding behavior**. GnIH also participates in the control of feeding behavior in birds and mammals. Central administration of GnIH increases food intake in birds and mammals. GnIH neurons project to neuropeptide Y (NPY) neurons and pro-opiomelanocortin (POMC) neurons. GnIH inhibits the firing rate in POMC neurons and has a predominantly inhibitory effect on action potential activity in NPY neurons. Thus, GnIH neurons may stimulate feeding behavior by inhibiting POMC neurons and NPY neurons in the hypothalamus. Future studies are needed to further develop the concept of central mechanism of GnIH actions on the control of feeding behavior. See the text for details.

In fact, intracerebroventricular (ICV) injection of GnIH stimulates food intake in chicks (132). Administrations of GnIH-RP-1 and GnIH-RP-2 also stimulate food intake in chicks (132). In further support of these findings, immunoneutralization of GnIH by central antiserum administration suppresses fasting-induced appetite, but does not modify feeding at *ad libitum* conditions in chicks (132). Similarly, ICV injection of GnIH, but not of GnIH-RP-1, stimulates feeding and suppresses plasma LH in adult Pekin ducks (133). Together, it is considered that at least GnIH is involved in the control of feeding behavior as well as reproduction in birds (Table 1; Figure 2).

To clarify the neurochemical cascade underlying GnIH actions on feeding behavior, Tachibana et al. (134) examined if GnIHs orexigenic effect occurs *via* the opioid and nitric oxide (NO) systems. According to Tachibana et al. (134), the orexigenic effect of centrally injected GnIH is attenuated by co-injection of an opioid μ-receptor antagonist β-funaltrexamine but not an opioid δ-receptor antagonist ICI-174,864 and an opioid κ-receptor antagonist nor-binaltorphimine in chicks. GnIH-induced feeding behavior is not affected by co-injection of a non-selective NO synthase inhibitor (134). More recently, McConn et al. (37) also investigated the mechanism of the orexigenic response of GnIH in chicks. ICV injection of chicken GnIH increases neuropeptide Y (NPY) mRNA but decreases pro-opiomelanocortin (POMC) mRNA in the chick hypothalamus (37). Additionally, ICV injection of chicken GnIH increases c-Fos expressed cells in the lateral hypothalamic area (LHA) (37). McConn et al. (37) further showed that in the isolated LHA, ICV administration of GnIH increases melanin-concentrating hormone (MCH) mRNA. Based on these findings, it is considered that opioid μ-receptor-positive neurons, and NPY, POMC, and MCH neurons are part of the orexigenic regulation by GnIH in birds.

In mammals, ICV administration of GnIH stimulates food intake in rats (44) and sheep (135). Additionally, food restriction activates GnIH neurons and GnIH infusion inhibits sexual motivation in hamsters (136, 137). GnIH mRNA levels are lower in male and female obese mice than wild-type animals, suggesting that GnIH stimulation of feeding circuits is reduced when energy storage is maximum (138). Furthermore, the inhibition of LH concentrations by food restriction is reduced in GnIH receptor (GPR147) knockout mice (139). Fu and van den Pol (140) reported in mouse brain slices that chicken and human GnIH inhibit POMC neurons and decrease Kiss cell excitation by opening potassium channels. Jacobi et al. (141) also reported in mice that GnIH inhibits POMC neurons’ firing rate and predominantly inhibits NPY neurons’ action potential activity as shown in Figure 2. Jacobi et al. (141) further reported that GnIH fibers have close contacts to NPY neurons.

Together, these results indicate that GnIH is involved in the control of feeding behavior in birds and mammals by the similar mechanisms (Table 1; Figure 2). Future studies are needed to further develop the concept of central mechanism of GnIH actions in the regulation of feeding behavior.

## GnIH Control of Reproductive Behavior

Gonadotropin-inhibitory hormone also acts in the brain to control reproductive behaviors including sexual and aggressive behaviors (28, 142, 143) (Table 1; Figure 1). First, Bentley et al. (142) reported that central administration of GnIH inhibits copulation solicitation of estrogen-primed female sparrows exposed to male song. There is evidence that GnRH2 enhances copulation solicitation of estrogen-primed female sparrows exposed to male song (144). GnIH neurons terminate in the close proximity of GnRH2 neurons and it was also shown that GnRH2 neurons express GnIH receptor mRNA in songbirds (35). Accordingly, GnIH may inhibit copulation solicitation by inhibiting GnRH2 neuronal activity in female songbirds (142). Subsequently, Ubuka et al. (143) investigated this possibility by testing how RNA interference (RNAi) of GnIH gene affects the behavior of male and female white-crowned sparrows. It was found that GnIH RNAi reduces not only resting time, but also spontaneous production of complex vocalizations and stimulates agonistic vocalizations. In addition, it was shown that GnIH RNAi increases song production of short duration when they were exposed to novel male songs in male birds. These findings indicate that GnIH gene silencing induces arousal in birds. Ubuka et al. (143) further found that the activities of the birds are correlated negatively with GnIH mRNA expression in the PVN. In female birds, GnIH RNAi decreases GnIH neuronal fiber density in the ventral tegmental area. Further, GnRH1 and GnRH2 neurons’ number, which receives appositions of GnIH neuronal fiber terminals, is correlated negatively with the activity of male birds (143). Recently, Ubuka et al. (28) have further demonstrated in male quail that GnIH inhibits aggressive behavior. It is thus becoming clear in birds that GnIH decreases sexual and aggressive behaviors [for reviews, see Ref. (24, 25, 115)] (Table 1; Figure 1).

In mammals, Johnson et al. (44) also reported that central administration of GnIH decreases male sex behavior in rats. On the other hand, there is a report showing that central administration of GnIH decreases sexual motivation and vaginal scent marking without affecting lordosis behavior in female hamsters (137). Piekarski et al. (137) showed that GnIH administration alters fos expression in the medial preoptic area (POA), the medial amygdala as well as the bed nucleus of the stria terminalis, key neural loci implicated in female sexual behavior. These findings suggest that GnIH is a modulator of proceptive sexual behavior and motivation in female animal (Figure 1). Accordingly, GnIH does not only control the HPG axis, but it may also modulate the neural circuitry underlying socially motivated behavior as in birds [see Ref. (26) for a review].

It is known that the interactions of neuropeptides and neurosteroids regulate brain functions [for a review, see Ref. (145)]. Recently, Ubuka et al. (28) discovered that GnIH activates cytochrome P450 aromatase (P450arom) and stimulates neuroestrogen synthesis in the quail brain (28) (Figure 1). Abundant GnIH immunoreactive neuronal fibers are distributed in the vicinity of P450arom immunoreactive cells in the POA (28). It was also shown that GnIH receptor is expressed in P450arom immunoreactive cells in the POA (28). Furthermore, GnIH increases neuroestrogen synthesis by stimulating P450arom activity through GnIH receptor in the POA (28) (Figure 1). Importantly, GnIH actions on neuroestrogen synthesis decrease aggressive behavior in birds (28) (Figure 1), providing a new finding that GnIH modifies neurosteroidal milieu in the brain to modulate aggressive behavior [see Ref. (146) for review]. Future studies are needed to develop the emerging concept of GnIH and other hypothalamic neuropeptides modifying the neurosteroidal milieu in the brain and the impact of its function.

## Conclusion

The discovery of GnIH in 2000 and the studies to understand its functions have advanced reproductive neuroendocrinology. It appears that GnIH acts on the pituitary and within the brain and modulates the reproductive axis as well as reproductive behaviors. Furthermore, recent studies have demonstrated that GnIH controls feeding behavior in vertebrates, such as birds and mammals. Thus, the last 15 years of GnIH research has led to a better understanding of the neuroendocrine control mechanism of feeding and reproductive behaviors as well as reproduction.

## Author Contributions

All the authors listed have made substantial, direct, and intellectual contribution to the work and approved it for publication.

## Conflict of Interest Statement

The authors declare that the research was conducted in the absence of any commercial or financial relationships that could be construed as a potential conflict of interest.
